# Reinforced lengthening Achilles tendon Z-plasty – ex vivo assessment of biomechanical augmentation with surgical-fiberlock technology

**DOI:** 10.1007/s00264-025-06481-9

**Published:** 2025-03-20

**Authors:** Thomas Dreher, Andrea Moehl, Elias Bachmann, Arend Nieuwland, Jess G. Snedeker

**Affiliations:** 1 Department of Pediatric Orthopedics, Balgrist University Hospital Zurich, University of Zurich, Zurich, Switzerland; 2 Department of Pediatric Orthopedics and Traumatology, University Children’S Hospital Zurich, Zurich, Switzerland; 3 Zurimed Technologies AG, Zurich, Switzerland; 4 Department of Orthopedics, Balgrist University Hospital, University of Zurich, Zurich, Switzerland; 5 Institute for Biomechanics, ETH Zurich, Zurich, Switzerland

## Abstract

**Purpose:**

Tendon lengthening is a common lower limb surgical procedure in paediatric orthopaedics and deformity correction. Healing of a lengthened tendon is typically supported by casting and unloading of the operated limb. Although tendon rupture or overcorrection may adversely affect surgical outcomes, few studies have examined surgical means of improving post-operative stability of the tendon. We aim to evaluate a novel method for augmenting Z-Plasty tendon lengthening as a first step to clinical translation.

**Methods:**

In this experimental ex vivo study, we employed a bovine flexor tendon model (*n* = 18) to examine a novel mechanical augmentation method after tendon lengthening by Z-plasty. Conventional surgical suturing of the imposed Z-plasty (*n* = 6) and an experimental group (*n* = 6), in which additional augmentation was performed by interlocking fibres of a biomaterial scaffold to the underlying tendon using a novel micro-needling technique, were compared to native tendons (*n* = 6).

**Results:**

The needle interlocked scaffold successfully augmented the suture repair, showing more than doubled ultimate failure force compared to controls (482 ± 107 N vs. 206 ± 37 N, *p* < 0.01), and more than 1.5-fold repair stiffness (41 ± 7 N/mm vs. 26 ± 9 N/mm, *p* < 0.01).

**Conclusion:**

We conclude that the use of an interpenetrating biomaterial scaffold represents a promising new approach for improving biomechanical tendon properties, which may have an implication on the stability of tendon suture, lengthening and tendon transfer procedures as well as on post-operative management and earlier mobilization.

## Introduction

Muscle–tendon shortness is a common problem when treating extremity deformities in children and adolescents. Tendon lengthening is frequent in lower limb surgical procedures in paediatric orthopaedics, especially in patients with cerebral palsy, residual or recurrence of club foot, as well as patients with Friedrich’s ataxia [1–5]. Several options for muscle–tendon lengthening are described, with Z-plasty being one of the most commonly used techniques [6–9], and a range of different suturing techniques have been used to secure Z-lengthening [10–16]. To protect the surgically imposed length of the tendon, postoperative tendon healing is typically load-protected by joint immobilization through use of a cast for at least four weeks, with full, partial or no weightbearing according to various expert recommendations [17–22]. The paediatric patient population, however, is known to have reduced compliance compared to the adult population. Especially toddlers and preschoolers cannot be expected to reliably and consistently perform partial weight bearing during the initial postoperative phase. Tendon load protection is done to avoid adverse effects such as acute tendon-suture rupture or sub-failure elongation of the tendon in the early phase of healing that can lead to suboptimal length of the healed muscle–tendon unit. Acute rupture commonly needs acute revision surgery, while chronic elongation may lead to persistent functional deficits related to gait performance such as crouch gait or loss of muscle force after tendon Achilles lengthening (TAL) [3, 23–27].

Despite the little evidential basis for an absolute need to immobilize a patient after tendon lengthening surgery, it is nonetheless the clinical standard of care. There are very few studies evaluating the ultimate tendon suture failure force after Z-plasty [28], and to the knowledge of the authors, there is no published Z-plasty suturing technique that could plausibly withstand immediate post-operative loading without irreversible tendon elongation. Earlier weightbearing and potential early mobilization without casting after Z-plasty would represent a substantial advancement in patient care. Toward this goal, the reduction of the risk for overlength after Z-plasty would be desirable. Augmentation methods for tendon repair after rupture are described in the literature [29–34]. A novel technique for tendon augmentation was recently described by Meyer et al. showing the advantages of additional tendon augmentation with the so-called “Surgical-Fiberlock” technology [35]. This method works by use of micro-needling to interpenetrate a non-woven biomaterial patch onto the underlying tendon tissue.

The purpose of the current study is to test mechanical stability of conventional Z-plasty sutures and to investigate whether biomechanical properties can be meaningfully improved by additional augmentation by using a surgical interlocking of a biomaterial patch atop the tendon suture.

## Materials and methods

This study uses mechanical testing to assess the performance of different surgical approaches to flexor tendon lengthening. For this study, bovine flexor tendons were used as model as they have been reported as being similar to children’s Achilles tendon in terms of suture retention characteristics including ultimate force and elongation under load [36, 37]. Bovine flexor tendons were obtained from a local abattoir (Metzgerei Angst, Zurich, Switzerland) and stored at −20 °C after dissection. The tendons were kept hydrated with phosphate-buffered saline (PBS) to maintain their handling and biomechanical properties. Once thawed, the tendons were cut to a length of 18 cm and pulled through a tendon diameter gauge (Karl Storz, Tuttlingen, Germany) which allowed diametral assessment within 0.5 mm precision. A total of 18 (n = 6 per group) tendons were prepared for biomechanical testing.

## Human and animal right statement

The study was performed in accordance with the ethical standards in the 1964 Declaration of Helsinki and in accordance with relevant regulations of the US Health Insurance Portability and Accountability Act (HIPAA). No further approval of the local ethical Review Committee was needed, as no live animal data were used. The bovine flexor tendons were obtained from a local abattoir.

### Group definition and surgical techniques

Each of 18 bovine tendons was randomly assigned to either the control group (*n* = 6) or one of the experimental groups (*n* = 6 per group). In the control group, tendons were longitudinally halved to reflect the mechanical properties of a single strand as created with the surgical technique of the Z-plasty. In the first experimental group, an isolated Z-plasty tendon lengthening was performed (Group ‘Z-Plasty only’), in the second experimental group the Fiber Lock PET-patch (FLP) augmentation was performed additionally to the tendon lengthening Z-Plasty (Group ‘Z-Plasty + FLP’).

**Fig. 1:**
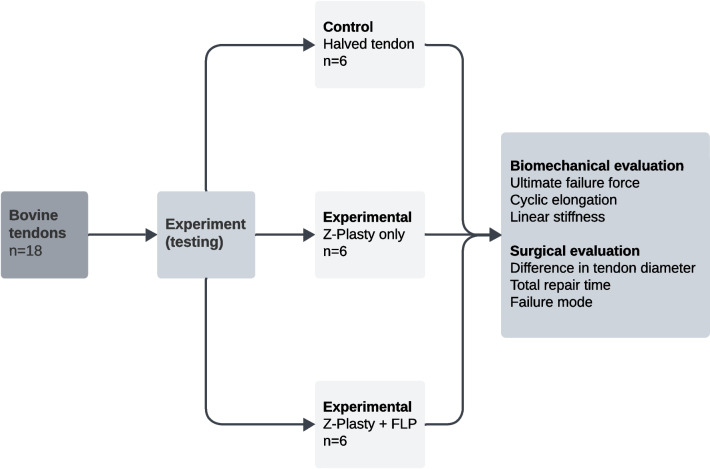
Study groups and surgical techniques

A senior consultant surgeon (T. D.) executed all repairs, preparation time was recorded for every specimen. All repairs were conducted on a tendon preparation board (Karl Storz, Tuttlingen, Germany), whereby the tendon ends were clamped and tensioned for the repairs. Two different surgical repairs were performed: the Z-plasty only approach and the patch augmented Z-plasty. Z-plasty was done by conventional surgical technique (Fig. 1 A): the longitudinal central Z-plasty incision measuring 5 cm was imposed and the construct was then lengthened by 2 cm. For all repairs, Vicryl Plus USP 1 CT-2 (Ethicon, Sommerville, NJ, USA) was used to first perform an augmentation of both strands with typical baseball stitches, then to suture the opposing arms of the Z-plasty tendon parts together. For the patch augmented Z-plasty (referenced as Z-plasty + FLP), the same Z-plasty was performed as previously described and then patch augmented as follows: a 2 cm × 6 cm polyethylene terephthalate (PET) patch (SpeedPatch® PET R, ZuriMED Technologies AG, Zurich, Switzerland) was wrapped around the repair site and clamped with an O-shaped surgical clamp (Fig. 1 B). The application instrument (FiberLocker Instrument R, ZuriMED Technologies AG, Zurich, Switzerland), which uses a reciprocating barbed blade to draw fibers from the patch directly into the underlying tendon, was used to attach the patch on top of the tendon with a frequency of 42 Hz and a duration of 17 s/cm^2^. This previously described method, coined by Meyer et al., as *“fiber interlocking”*, interpenetrates individual synthetic fibres of the non-woven patch within collagen fibres of the native tendon tissue, resulting in a strong interlaced interface[38] without additional suture material. The clamp and consequently fibre interlocked fixation was repositioned three times on both sides of the tendon to cover the entire patch area. Also, the thickness of each specimen after the repair was measured with the tendon diameter gauge.

### Biomechanical testing

**Fig. 2:**
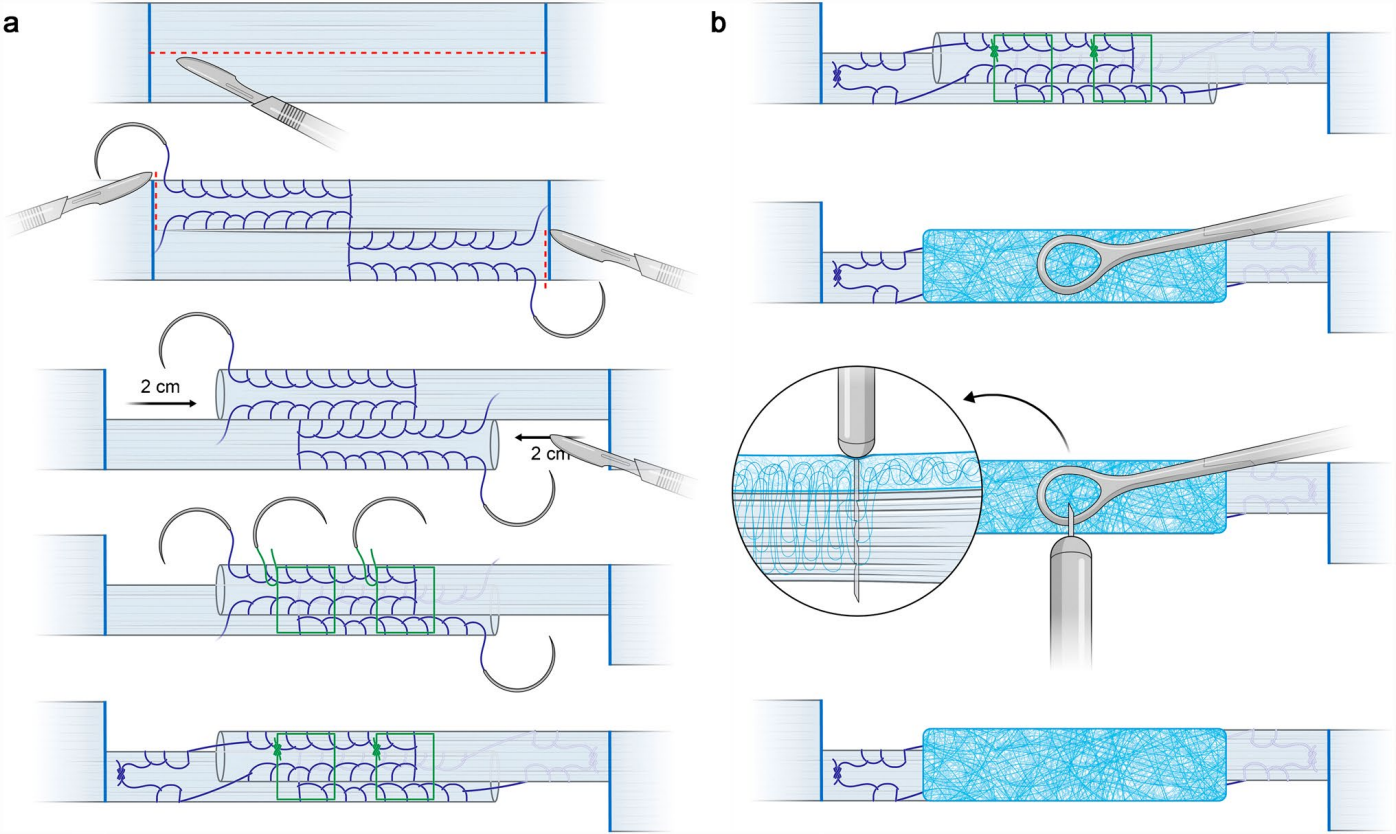
**A** Procedure for Z-plasty and **B** Z-plasty with PET patch (FLP)

A universal material testing machine Z010 (ZwickRoell GmbH, Ulm, Germany) with a 10 kN load cell was used for testing and data was recorded with the manufacturer’s software (testXpert III ZwickRoell, Ulm, Germany). Specimens were mounted between two axial aligned tendon clamps with a defined initial grip-to-grip separation of 100 mm (Fig. 2). Mechanical testing protocols in literature vary greatly for investigating Achilles tendon repairs, however, a protocol that has previously been used in studies focusing on early mobilization testing of Achilles tendon repairs was chosen as the most applicable [29, 31–33]. We consequently applied a pretension of 10 N, followed by cyclic loading for 40 cycles from 2 to 30 N under load control at a rate of 5 N/s and ultimately followed by a load to failure testing at 2 mm/s.

The cyclic loading outcome measure included elongation during cyclic loading. The cyclic elongation was determined using the standard travel measurements of the material testing machine. The delta in travel between the local maxima of the first and 39th cycle was used to calculate this parameter. The tensile testing outcome measures were ultimate failure force and linear stiffness (with stiffness values extracted from the load-deformation curve between 30 and 120 N of applied force). Additionally, the failure mode was recorded for every specimen.

### Statistical evaluation

For statistical analysis, GraphPad Prism 9.5.1 (GraphPad Software, San Diego, CA, USA) was used. The Shapiro–Wilk test was applied to evaluate normality. A post-hoc power analysis using RStudio version 2021.09.1 (R Studio, Boston, MA, USA), focusing on the ultimate failure force parameter. The analysis indicated that a minimum sample size of two samples per group was required.

When comparing three groups for the biomechanical outcome parameters, a Brown-Forsythe test was used to test the equality of standard deviations. An ordinary one-way analysis of variance (ANOVA) was chosen as the parametric test and the Kruskal–Wallis test as the non-parametric test. For the parametric test with unequal variances, Welch’s ANOVA was used. For evaluating multiple comparisons, Tukey’s multiple comparisons test was applied for the parametric test, while Dunn’s multiple comparison test for the non-parametric test, and Dunnett’s T3 multiple comparison test for the parametric test with unequal variances.

In the statistical evaluations of the two groups (unpaired) regarding the surgical time and the tendon diameter change, an F-test was used to test the equality of standard deviations. Mann–Whitney test was chosen as the non-parametric test and unpaired t-test as the parametric test. For a parametric test with unequal variances, the unpaired t-test with Welch’s correction was used.

Level of significance was set to *p* < 0.05 for all tests.

## Results

**Fig. 3:**
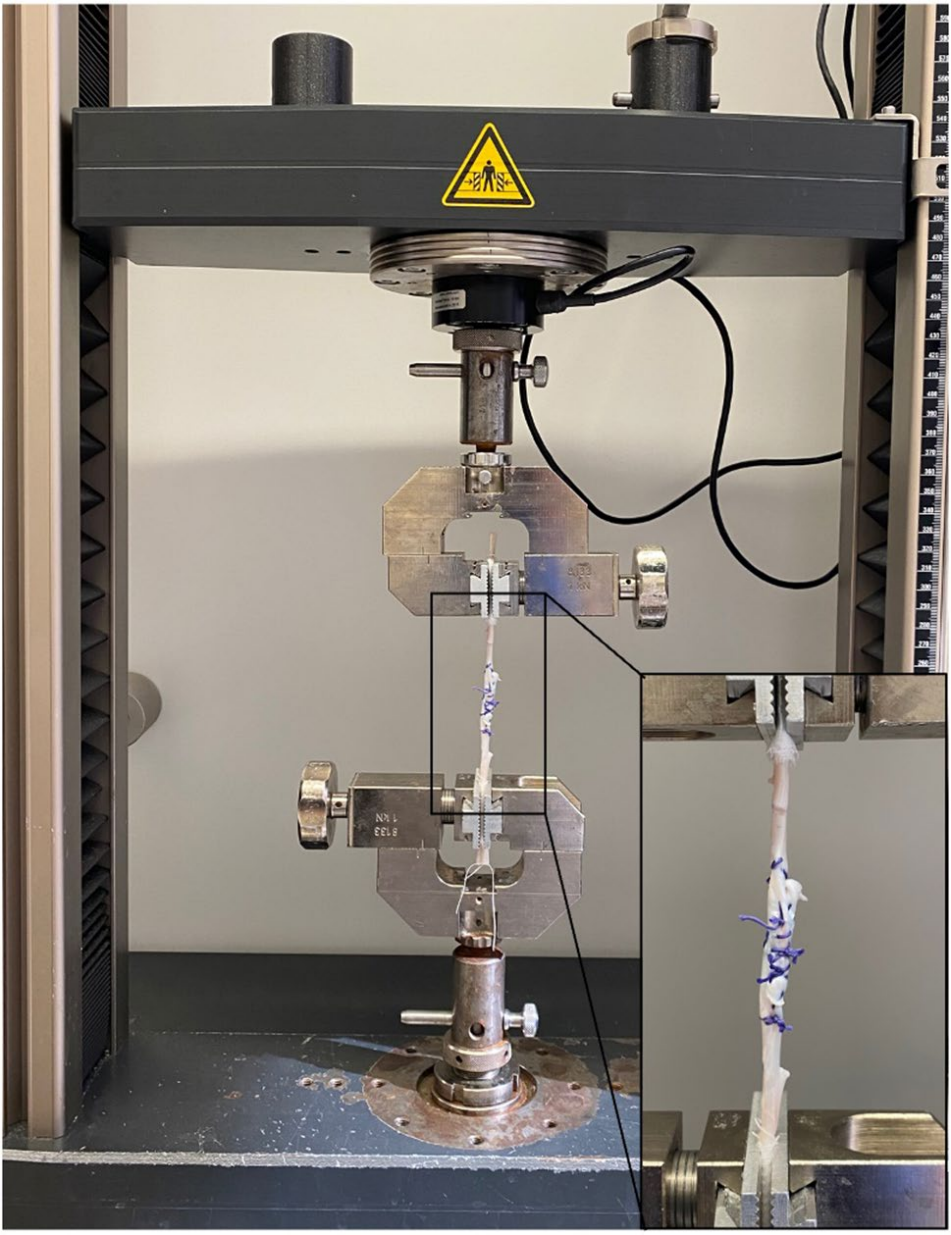
Set up of tensile testing to failure with conventional Z-plasty specimen

**Fig. 4:**
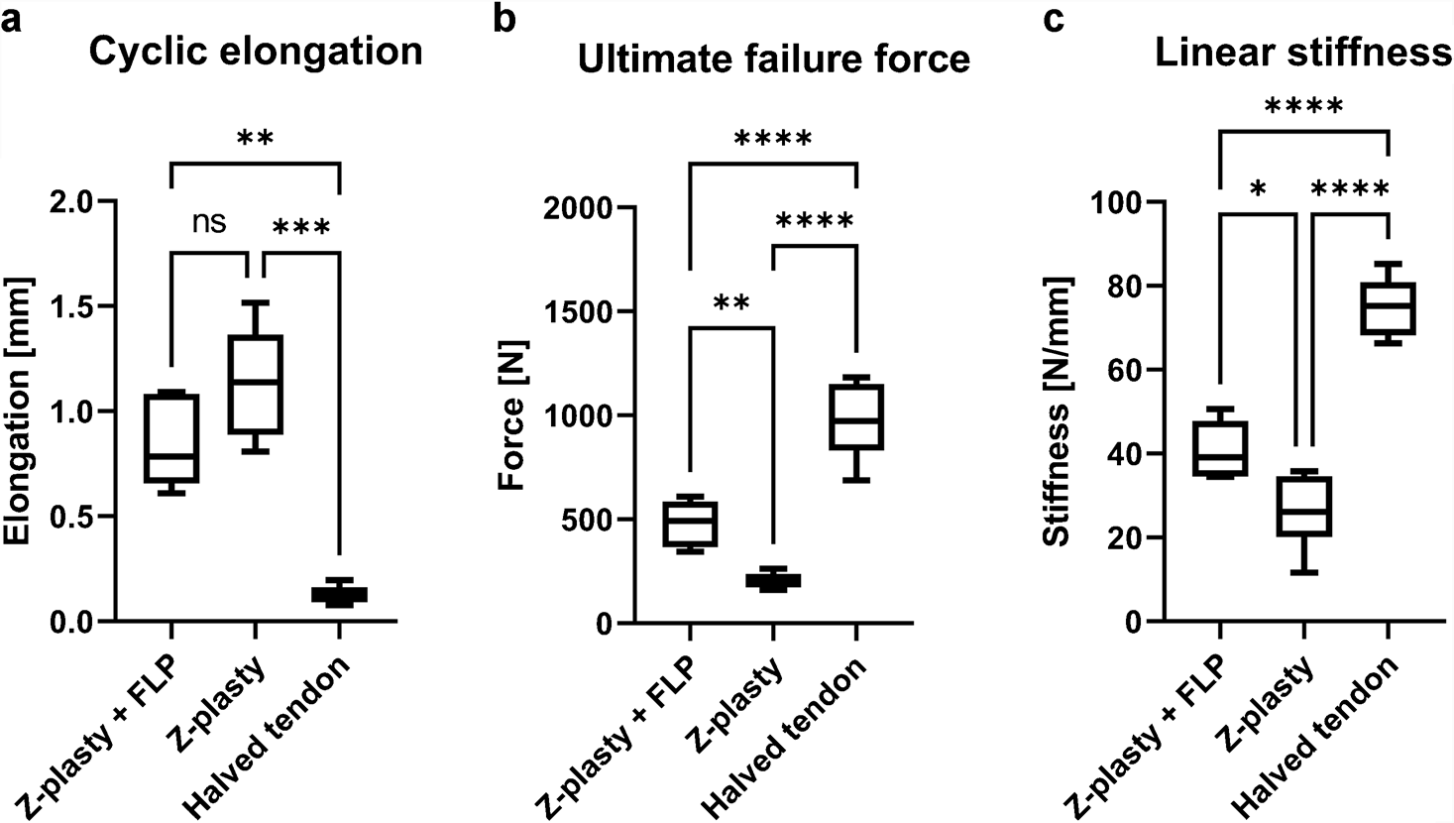
Boxplots with median and whiskers from minimum to maximum for pull-to-failure testing **(A)** Cyclic elongation **(B)** Ultimate failure force **(C)** Linear stiffness

Control specimens showed mean ultimate failure force of 973N. Conventional Z-plasty resulted in 206N (21% of reference), whereas patch augmented Z-plasty achieved 482N (50% of reference). Cyclic elongation showed significant difference among both surgical groups compared to control (W = 67.29, *p* < 0.01) with the Welch’s ANOVA test, while ultimate failure force (F = 60.02, *p* < 0.01) and linear stiffness (F = 65.86, *p* < 0.01) also significantly differed among the groups tested with ANOVA (Fig. 3). Ultimate failure force for the Z-plasty + FLP group was increased by 134% compared to the Z-plasty group and linear stiffness was 58% higher compared to the Z-plasty group (Fig. 4).

Outcome measures based on the surgical technique can be found in Table 1 and were only evaluated for the two groups requiring the application of a surgical intervention. The outcome parameters regarding the difference in tendon diameter and total repair time were non-parametric and therefore evaluated with a Mann–Whitney test. The Mann–Whitney test demonstrated a significant difference between the two surgical groups regarding the difference in tendon diameter (p = 0.0108) but not in total repair time (p = 0.2987). Finally, the failure mode of each specimen was defined and classified according to suture cut through and tendon rupture as distinct failure modes (Table 2).

**Table 1:** Outcome parameters of surgical technique (median (interquartile range (IQR)))

	**Z-plasty**	**Z-plasty + FLP**
Difference in tendon diameter [mm]	1.0 (0.5–1.1)	1.5 (1.5–2.0)
Total repair time [s]	550 (544–618)	613 (573–627)

**Table 2:** Failure modes distribution among groups

	**Suture cut-through**	**Medial tendon tear/ tendon rupture**
Z-plasty (n = 6)	5/6	1/6
Z-plasty + FLP (n = 6)	3/6	3/6

## Discussion

Although of central concern to surgeons, the literature on biomechanical properties after Z-plasty by standard suturing is sparse. We used a bovine model mimicking tensile and elastic properties of the human Achilles tendon[36, 37] to show that microneedle-based interlocking of a patch to augment an underlying suture repair can substantially improve biomechanical resistance of Z-plasty to failure and elongation. Ultimate failure force and linear stiffness were both relevantly improved by use of a novel technique based on interlocking fibres of a PET patch to achieve a mechanical augmentation of the underlying suture-tissue interface, without clinically relevant increases in total tendon diameter (0.5 mm increase of patch augmentation compared to Z-plasty only) or total surgical repair time compared to the suture-only control group. Especially compared to halved tendon strands inherent to standard Z-plasty, substantial clinical and statistical effects are noted comparing PET patch augmentation to conventional surgery.

The used PET patch is a microporous, non-resorbable surgical mesh made of 100% PET, a material that is known for its high tensile strength and durability. PET has a well-established history in orthopaedics[39–42], and its clinical application in nonwoven structures, which support tissue integration, has been explored in various animal models[43, 44] and human studies[45, 46]. Among others, Kimura et al. reported a relevant fivefold improvement of tensile strength of the tendons on middle to long term healing, gradually increasing from 61 N ultimate failure force directly postoperative to 306 N 12 weeks postoperative. Ultrasound documentation showed robust fibrous tissue in growth in the felt patch framework [44]. Similarly, Meyer et al. demonstrated in an in-vivo study in sheep infraspinatus tendon a non-inflammatory host tissue response to the used nonwoven PET patches with neovascularization and new tissue formation throughout the patch [35]. Although the indication presented in this manuscript differs, as the same PET patch and application device are used, a similar host tissue response is anticipated when used for Achilles tendon repair augmentation. The inflammatory reaction observed in the sheep model was minimal and appeared to plateau after the early phases of healing. [35]. While neovascularization and new tissue formation were evident within the PET patch, the extent of fibroblastic activity and potential adhesion formation appeared limited in this model. Postoperative changes and inflammatory based remodelling of the tendons several weeks after surgery can, due to ex vivo nature of the study, currently not be assessed. Long term effects are to be evaluated in further in vivo experiments essential to evaluate the biological integration and mechanical behavior of patch-augmented repairs over time, particularly under conditions mimicking daily ambulation. These studies should also assess the capacity of this biomechanical augmentation to support earlier and safer weight-bearing.

The currently measured improvements in ultimate failure force are consistent with allowing substantially higher post-operative load bearing than with the mean ultimate failure force of 482 N corresponding to 49 kg of weight bearing. In the scope of paediatric orthopaedic surgery, this could reasonably imply potential for meaningful surgical prevention of adverse effects such as acute tendon suture rupture or overcorrection due to elongation of the tendon in the early phase of healing that can adversely affect the functional outcome for the patient. Considering the measured standard deviation of 107 N, weight bearing mobilisation of up to approximately 38 kg (375 N) could safely be born. Clinically, this could mean less restrictive and even (close to) normal, postoperative mobilisation at loading levels compatible with no use of casting or partial weight bearing. Especially in younger patients, unable to adhere to weight bearing limitations, both physically as well as mentally, this would represent a dramatic improvement of current postoperative rehabilitation regimes. However, since this study is limited to an experimental setup, the transfer of the new insights into clinical practise needs further documentation and analysis.

The bovine tendons used in this study have a smaller cross-sectional area compared to human Achilles tendons, making direct scaling of the measured forces to human clinical scenarios challenging. However, the mechanical properties of bovine tendons, such as stiffness and failure force, provide a relevant proxy for preclinical testing. While direct extrapolation is not feasible, the observed biomechanical advantages of patch-based augmentation, particularly the doubling of repair strength, remain clinically significant. Static testing was deliberately chosen as a foundational approach to evaluate the biomechanical properties of the patch-augmented repair under controlled conditions. This provides critical baseline data to guide the design of future in vivo and dynamic studies, which will address the complexities of cyclic loading and tendon remodeling.

Dynamic loading, such as during walking or running, introduces additional factors like cyclic fatigue, strain-rate dependence, and viscoelastic effects, which were not accounted for in this static testing. These nuances highlight the need for future studies to address whether the observed improvements under static conditions translate to dynamic performance.

Optimal tendon stiffness plays a key role in facilitating effective muscle–tendon interactions.

Both after tendon rupture with conservative treatment and after tendon repair surgery, clear reductions in tendon stiffness are observed and may lead to alterations in muscular balance, gait pattern and return to sports [47]. The observed higher linear stiffness of the augmented tendon, with on average 1.5-fold higher stiffness in the fiber interlocked patch augmented Z-plasty compared to conventional Z-plasty and more than halve the linear stiffness of tendons without further surgical manipulation, is not likely to directly influence the postoperative mobilisation scheme. As higher tendon stiffness shows clinically better performance in athletes[48, 49], the improved stiffness after tendon augmentation could be postulated to translate to better long-term outcomes and improved tendon function compared to standard suture only Z-plasty repairs. As no clear cut off values for tendon stiffness regarding optimal tendon performance have been defined, and as middle to long term histological and biomechanical analysis during the tendon regeneration process are, due to the ex vivo nature of the experiment, not available, we are careful not to overinterpret the current results.

The current results do establish increased stiffness in the vulnerable early postoperative phase of the rehabilitation, therefore reducing the likelihood of occurrence of adverse events (i.e. unplanned lengthening).

Regarding tendon elongation, both conventional Z-plasty and augmented Z-plasty techniques perform comparable in a static environment as tested, showing no statistically significant differences between both repair techniques. As Delp et al. showed in their work, during midstance phase of gait, the maximum isometric moment generated by soleus varies greatly depending on the performed tendon lengthening. A 30% decrease in maximum isometric moment occurs after 1-cm tendon lengthening compared to 85% decrease after 2-cm tendon lengthening[27]. Dietz et al. discussed the high rate of over weakening of the triceps surae after Achilles lengthening surgery (defined by the need for a floor reaction brace) for equinus of the ankle in ambulatory patients with cerebral palsy[23]. These works underline that tendon lengthening after repair can critically impact the force–length relationship of the muscle–tendon unit. Changes in tendon length may alter muscle function, affecting strength, control, and overall biomechanical efficiency. Although our current analysis was performed in a static environment, the absence of significant elongation in the repaired tendons under these conditions is a promising indicator of repair stability.

This study is the first ex vivo analysis to explore use of fiber interlocking principles for lower limb tendon repair. Under the controlled settings of the current laboratory study, certain physiological mechanisms cannot be accounted for, including continuous over tensioning of load-bearing tendons during normal ambulation. Similarly, the use of an animal model presents limitations, and translating these results to human clinical application must be done with care. Nonetheless, the bovine tendon model demonstrates a high degree of mechanical and physical similarity to human tendons, and we assess any potential bias in this regard to be small. Finally, since ex vivo biomechanical testing protocols cannot faithfully represent tendon loading in living subjects, in vivo animal studies are a logical and necessary next step. The original study describing the fibre interlocking principles[35] shows clear benefits of patch augmented suture repairs compared to conventional suture repairs regarding suture retention strength without detectable difference in repair stiffness: a nearly doubled load to failure was demonstrated with the suture-free PET-patch augmentation to a suture-based tendon repair. These results illustrate the biomechanical improvement over current techniques, while reducing the likeliness of musculotendinous retraction after tendon repair.

Despite these limitations, we consider that surgically interlocked patch augmentation clearly holds promise for substantially improving the primary biomechanical performance of Z-plasty. Further improvements can be made regarding the surgical technique, potentially improving upon current biomechanical results. Fiberlock patch augmentation did not require meaningful differences in preparation time compared to conventional augmentation techniques. Improvements in technical efficiency over time can be expected as user experience is more extensive. The application of PET-patch augmentation does not significantly impact the conventional surgical workflow, suggesting that the technique is broadly applicable to tendon repair, tendon lengthening procedures using alternative techniques, and tendon-to-tendon transfers.

The results described here provide an important basis for future studies, both using preclinical in vivo models and for on label clinical use in humans. Fiber interlocking of a suture-free PET patch to augment Z-plasty demonstrates biomechanical superiority over standard suture-based Z-plasty (control group) with significantly higher ultimate failure force and linear stiffness. The ability of a fiber-interlocked patch augmentation to nearly double tendon suture strength, may potentially enable early mobilisation of (paediatric) patients, possibly expanding to cast-free and/or full weight bearing ambulation and thus represents a very promising new approach to tendon surgery in general for translation through in vivo experiments into clinical practice.

## Data Availability

The authors confirm that the data supporting the findings of this study are available within the article and its supplementary materials.

## Declarations

The authors declare that no funds, grants, or other support were received during the preparation of this manuscript.
